# A method for acquiring random range uncertainty probability distributions in proton therapy

**DOI:** 10.1088/1361-6560/aa9502

**Published:** 2017-12-18

**Authors:** S M Holloway, M D Holloway, S J Thomas

**Affiliations:** 1 Department of Oncology, University of Cambridge, Cambridge, United Kingdom; 2 Department of Medical Physics and Biomedical Engineering, University College London, London, United Kingdom; 3 British Antarctic Survey, Cambridge, United Kingdom; 4 Department of Medical Physics, Cambridge University Hospitals, Cambridge, United Kingdom; 5 Author to whom any correspondence should be addressed.; s.holloway@ucl.ac.uk

**Keywords:** radiotherapy, proton beam therapy, IGRT, treatment planning, range uncertainty

## Abstract

In treatment planning we depend upon accurate knowledge of geometric and range uncertainties. If the uncertainty model is inaccurate then the plan will produce under-dosing of the target and/or overdosing of OAR. We aim to provide a method for which centre and site-specific population range uncertainty due to inter-fraction motion can be quantified to improve the uncertainty model in proton treatment planning.

Daily volumetric MVCT data from previously treated radiotherapy patients has been used to investigate inter-fraction changes to water equivalent path-length (WEPL). Daily image-guidance scans were carried out for each patient and corrected for changes in CTV position (using rigid transformations). An effective depth algorithm was used to determine residual range changes, after corrections had been applied, throughout the treatment by comparing WEPL within the CTV at each fraction for several beam angles.

As a proof of principle this method was used to quantify uncertainties for inter-fraction range changes for a sample of head and neck patients of $\Sigma=3.39$ mm, $\sigma = 4.72$ mm and overall ${\rm mean} = -1.82$ mm. For prostate $\Sigma=5.64$ mm, $\sigma = 5.91$ mm and overall ${\rm mean} = 0.98$ mm. The choice of beam angle for head and neck did not affect the inter-fraction range error significantly; however this was not the same for prostate. Greater range changes were seen using a lateral beam compared to an anterior beam for prostate due to relative motion of the prostate and femoral heads.

A method has been developed to quantify population range changes due to inter-fraction motion that can be adapted for the clinic. The results of this work highlight the importance of robust planning and analysis in proton therapy. Such information could be used in robust optimisation algorithms or treatment plan robustness analysis. Such knowledge will aid in establishing beam start conditions at planning and for establishing adaptive planning protocols.

## 1. Introduction

There are many sources of range uncertainty in proton therapy, including both the systematic and random components arising from the uncertainty in Hounsfield Units (HU), dose calculation algorithms, daily setup, target delineation and organ motion (McGowan *et al* 2013). The range uncertainty problem may be relatively new to radiotherapy, but the uncertainty problem is not. Safety margins are grown based on known systematic and random uncertainties inserted into a margin recipe to ensure minimum target coverage, most notably van Herk’s PTV margin recipe (ICRU 1993, 1999, van Herk *et al* 2000, ICRU 2010). Newer planning techniques can be in conflict with several of the underlying assumptions taken in van Herk’s original margin design (Bohoslavsky *et al* 2013). As a consequence, the indirect estimation of the confidence level becomes unreliable. Intensity modulated particle therapy (IMPT) is one such example, where complex and inhomogeneous dose distributions are delivered, often with steep dose gradients. Along with the extra degree of freedom in the proton range, this renders the PTV an inadequate planning tool. This is partly due to the shift invariance assumption, as it cannot be applied to the proton case, and due to matching non-homogenous dose contributions from different beam angles within the target itself. The limitations of the PTV in complex proton cases have been addressed directly by several authors (Unkelbach *et al* 2007, Pflugfelder *et al* 2008, Fredriksson *et al* 2011, Chen *et al* 2012, Liu *et al* 2012) by developing probabilistic and robust optimisation algorithms for use in radiotherapy. Through using these methods, the treatment planner is no longer responsible for adding margins. Instead, the TPS builds its own *margins* into the dose distribution, governed by probabilistic planning criteria or a lower boundary with a predefined probability of occurring (Gordon *et al* 2010). These approaches are the correct way to deal with uncertainties when planning complex proton therapy treatments compared to current planning methods. However, many clinical proton therapy centres still use simpler techniques, such as the single field uniform dose (SFUD) approach (Perkó *et al* 2016). This is due in part, to a lack of understanding of these methods and the challenges involved in changing practices, as well as a need to fully understand range uncertainties. PTV-based and probabilistic/robust plans are equally dependent on sufficiently accurate knowledge of the distribution of the geometric uncertainty (Gordon *et al* 2010). In proton therapy this statement is equally true of the range uncertainty. Unkelbach *et al* (2007) suggested that for future studies tighter bounds on the actual magnitude of range uncertainty could be added with the aim to derive more precise uncertainty models for specific tumour sites and planning protocols.

The quantification of range uncertainties is required as input into robust or probabilistic optimisation (Unkelbach *et al* 2007, Gordon *et al* 2010) and for robustness analysis. Systematic setup uncertainties have shown to be measured for individual centres and specific treatment sites as described by Bolsi *et al* (2008) and random setup uncertainties using two different online matching methods were investigated by Liebl *et al* (2014) in the head and neck patients, however, using only rigid shifts and rotations. More accurate ways of determining systematic range error at HU calibration through use of dual energy CT (DECT) have been discussed (Bär et al^2017^). However, there is still a challenge in quantifying the random range uncertainty and the associated probability arising from patient motion and organ deformation throughout treatment. Currently, robust optimisation and analysis has been modelled relying on rigid isocentric shifts in CT data sets or from uniform percentage changes in the HUs (Albertini *et al* 2011, McGowan *et al* 2015, Lowe *et al* 2016). Despite ever more complicated methods of analysing plan robustness (Perkó *et al* 2016), which state the relevance of inter-fraction uncertainties, they do not include them or suggest a method for incorporating this error into the systematic range error.

In this paper we propose a method of quantifying the residual range error from inter-fraction motion, after online IGRT based corrections have been applied to create a population probability distribution of water-equivalent range changes in the CTV. This method is here applied to a set of daily TomoTherapy MVCT data from previously treated patients for two anatomical locations, prostate and head and neck, using several example beam directions as a proof of principle. Though using daily IGRT to quantify, or identify, range errors is not new (Bentefour *et al* 2015, Veiga *et al* 2016, Wang *et al* 2016), our results demonstrate how such a method could be used to acquire probability distributions of the residual range uncertainty. Future work will be to apply this method to CBCT and MRI using clinical proton plans and deformable image registration, with the vision that as the centre treats prospective patients a the model will be updated.

## 2. Methods and materials

### 2.1. Imaging modality

TomoTherapy is an integrated helical IMRT and IGRT system and provides volumetric, fan-beam megavoltage (MV) CT imaging on the treatment couch (Langen *et al* 2005). The clinical MVCT data was used along with the daily rigid couch shifts recorded directly by the TomoTherapy system.

### 2.2. MVCT calibration

The relationship between HU and electron density for the Addenbrooke’s MV imager was calculated by using the TomoTherapy ‘Cheese’ phantom (Langen *et al* 2010) with density plugs inserted. A shift in the HU representing water has a larger impact than a shift in the HU representing bone since a typical patient image contains more water equivalent density material than bone-like materials. For this reason real water has been used in the HU calibration. The density plugs provided have values specified in g ${\rm m}^{-2}$. To convert the TomoTherapy HU to relative proton stopping power the approximate values for the electron density relative to water for the density plugs were acquired directly from Gammex (Gammex 2009), these were interpolated for values required. These values were then converted to relative proton stopping power using the ratio shown in equation (1) (Schaffner and Pedroni 1998) and an energy-independent *β* value, where $I_{I}$ and $I_{w}$ are the ionisation potentials of tissue and water respectively, $I_{w}$ has been taken as 75 eV (ICRU 1993). The approximate mean excitation values, I-values, were supplied by Gammex (Gammex 2009).

1 \begin{align} \newcommand{\e}{{\rm e}} \displaystyle \label{eq1} S_{\rm rel} = \frac{\rho_{e}}{\rho_{ew}} \cdot \frac{\left[\ln \left(\frac{2m_e c^2 \beta^2}{I \cdot (1-\beta^2)}\right) - \beta^2\right]}{\left[\ln \left(\frac{2m_e c^2 \beta^2}{I_w \cdot (1-\beta^2)}\right) - \beta^2\right]} \nonumber \end{align}

2 \begin{align} \newcommand{\e}{{\rm e}} \displaystyle \label{eq2} R= \alpha \cdot E^{P}_{0}. \nonumber \end{align}

The absolute stopping power is clearly energy dependent from the Bethe–Bloch formula ($\beta=\frac{v}{c}$). However, it was calculated using the single range-energy relationship shown in equation (2) (Bortfeld and Schlegel^1996^), that the percentage difference in relative stopping power for the cortical bone insert for a 90 MeV proton beam and a 310 MeV beam was less than 0.6% for the two worst case energies used, 90 MeV and 310 MeV. With an error on the *β* value less than 0.6%, energy independence was assumed for this work. The calibration used is shown in table 1.

**Table 1.** Table of density inserts used to calibrate the MVCT and their corresponding physical densities, estimated electron densities and proton stopping powers relative to water as calculated from the Bethe–Bloch equation.

Tissue substitute	Density	e- Density	$SP_{w}$	HU
Air	0	0	0	$-970$
Lung	0.49	0.48	0.481	$-479$
Water	1	1.00	1.00	9.5
inner bone	1.139	1.09	1.081	118
Bone mineral	1.152	1.10	1.091	127
30% CaCO_3_	1.334	1.27	1.258	292
50% CaCO_3_	1.562	1.47	1.431	474
Cortical bone	1.824	1.69	1.622	673

### 2.3. WEPL calculation

The 3D MVCT data and planning structure sets were shifted in magnitude and direction as was applied by the radiographers clinically on set. The WEPL within the CTV was calculated using an effective depth algorithm adapted from Thomas *et al* (2011). In this study only co-planar beam angles were simulated so ray tracing was limited to the CT plane. For each angle, the ray from each of pixel was traced towards the beam focus, with the Hounsfield unit at each point converted to proton stopping power as described above. The summation of the values was multiplied by the step length in each pixel to give the effective depth at each point.

### 2.4. Patient sample

Nine prostate and seven head and neck patients were randomly selected, each of whom had consented for the use of their data for research. The standard protocol is to image daily using MVCT with a slice thickness of 6 mm for prostate and 3 mm for head and neck. For the prostate cases, six of the patients were on the PIVOTAL trial (Harris *et al* 2011). All patients, prostate and head and neck, were enrolled in the VoxTox study (VoxTox 2014). The bladder filling protocol for prostate patients was 3 cups of water 20 min before treatment. For both treatment sites daily imaging had been carried out using MVCT on TomoTherapy and rigid shifts applied after soft tissue matching has been carried out on set by the treatment radiographers.

### 2.5. Planning

In this study, dose was not calculated, however, in the case of the prostate data two clinically relevant beam angles have been used to investigate inter-fractional changes in WEPL; a coplanar anterior beam, and a lateral beam (0° and 90°). In the case of the head and neck coplanar beam angles of 0°, 25°, 70°, 110°, and 180° have been used. The use of these beam angles was to gain an insight into how increasing amounts of traversing material affects the magnitude of range error.

### 2.6. Imaging artefacts

**Figure 1.**
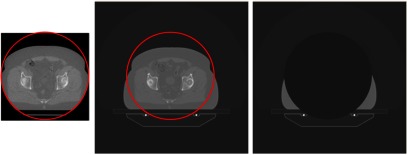
Example of Truncation correction for Prostate case. From left to right: MVCT image showing TomoTherapy scanning circle, planning CT showing TomoTherapy scanning circle, and finally, the mask used for this slice of the patient CT—figure reproduced from Thomas *et al* (2016) © 2016 The Authors. Published by the British Institute of Radiology. CC BY 4.0.

In prostate cases sometimes for larger patients a small volume of the body outline will be truncated. To correct for truncation artefacts due to the small FOV of the TomoTherapy imaging detector, which has a diameter of 38.6 cm, the planning kVCT was used with daily shifts applied to contribute data that existed outside of the TomoTherapy scanning circle. The truncated region of the body is fairly homogeneous so a uniform density mask was applied to the kVCT data as shown in figure 1. This assumption eliminated the need for a kVCT calibration. Though it may look like a lot of patient image data is being truncated, this occurs on a couple of slices for a couple of patients and it is only the tissue on the lateral beam direction directly in line with the prostate that is of concern. MVCT slices in the shoulder region for head and neck patients were excluded from the analysis, to avoid potential errors being introduced from a truncation artefact.

### 2.7. Range error calculation

The range error from inter-fraction motion was calculated by comparing the WEPL to each pixel within the CTV at each fraction to the equivalent pixel in a fraction chosen as a reference. The reference fraction, would be the first usable fraction, in most cases this was first fraction. Ideally, each treatment fraction would be compared to the planning CT, as this is the reference scan the treatment will be planned to. Due to the difference in the two modalities, kV and MV imaging, this has not been carried out. By taking the difference in WEPL between a reference fraction and consecutive fractions, an indication of inter-fraction range uncertainty for a given set of beam directions was obtained.

### 2.8. Statistics

In treatment planning a single composite range uncertainty is convenient for either choosing margins, or for using in a robust optimisation Yang *et al* (2012). To this end, estimates of the overall inter- fraction mean, random and systematic range error for all patients have been calculated. These have been calculated using methods similar to those set out by Greener (2003); weighting has been by number of images analysed rather than number of patients for both the overall mean and the systematic uncertainty (Σ). The systematic inter-fraction error was calculated by plotting a histogram of the mean range changes for each usable fraction across all patients and beam angles and taking the standard deviation. For prostate and head and neck patients, the standard deviations (*σ*) were calculated from all the non-binned fraction data, this means that the random uncertainty was calculated with weighting on number of data points and not number of images.

## 3. Results

**Figure 2.**
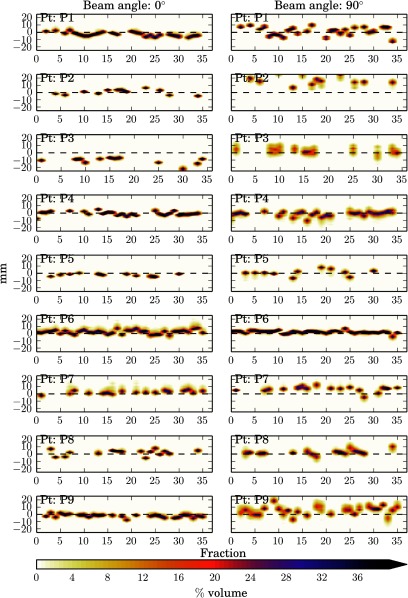
Heat plots of %volume range error histograms for prostate patients (in order top to bottom) with fraction using 0° and 90° beam angles.

**Figure 3.**
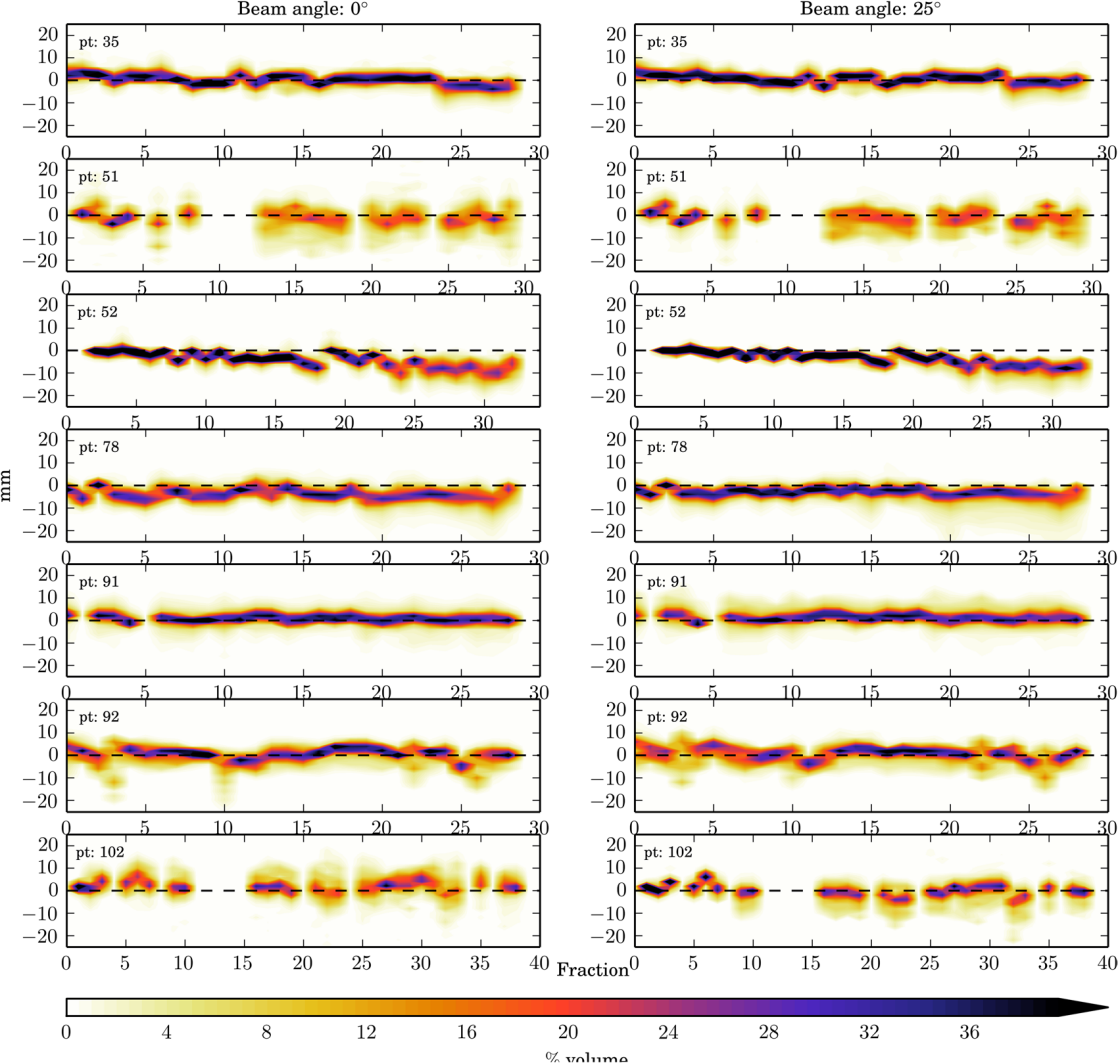
Heat plots of %volume range error histograms for all head and neck patients with treatment fraction at 0° and 25° beam angles.

**Figure 4.**
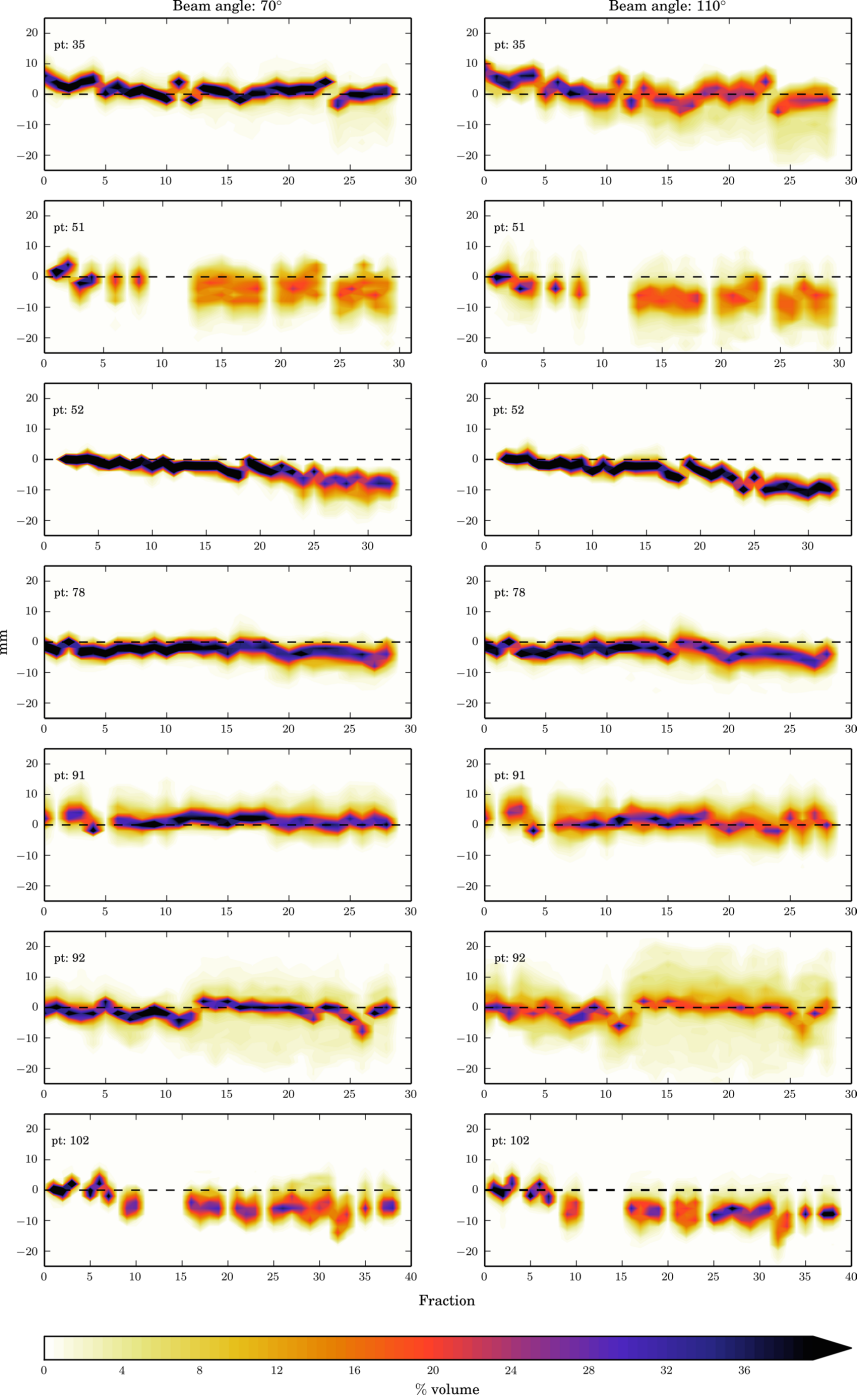
Heat plots of %volume range error histograms for all head and neck patients with treatment fraction at 70° and110° beam angles.

**Figure 5.**
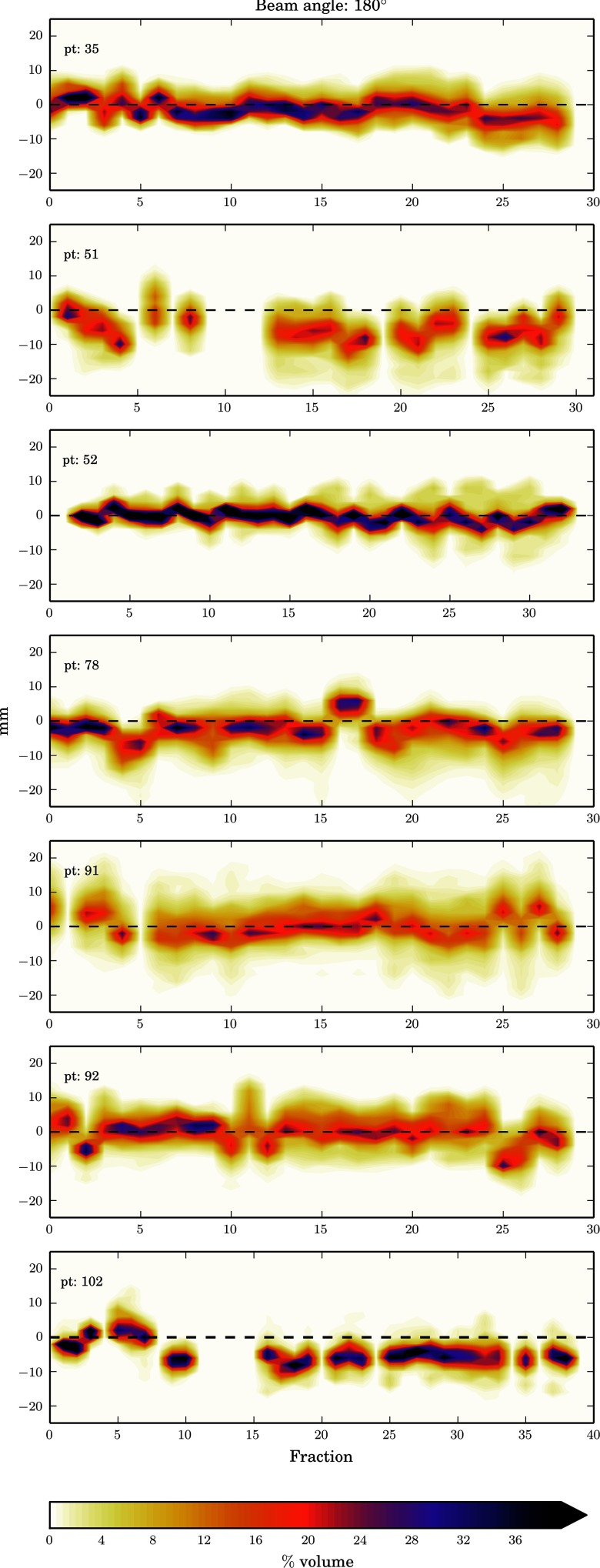
Heat plots of %volume range error histograms for all head and neck patients with treatment fraction at 180° beam angle.

The data has been presented as heat maps of the frequency of the WEPL difference at each fraction i.e. the darker the colour the greater the frequency of that magnitude of WEPL change. Figure 2, and figures 3–5, are the prostate and head and neck data respectively with each column representing a different beam angle and each row a different patient. Along the *x*-axis is time in terms of fractions, and along the *y*-axis is the change in range in millimetres. The zero line is shown so it can be seen how the mean range error deviates from zero throughout treatment. The plots have been truncated for visual purposes, but all data are included in the statistical analysis.

**Figure 6.**
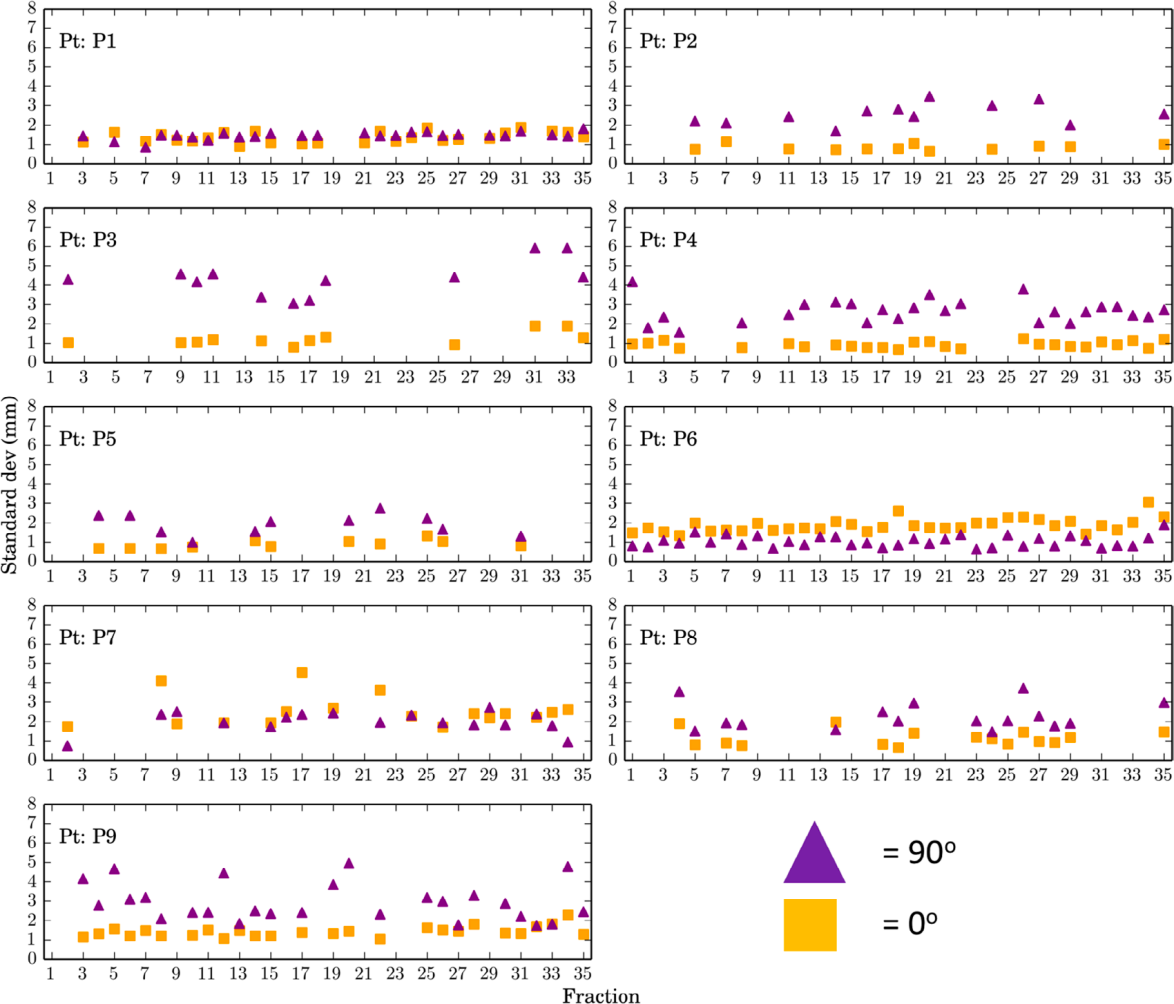
Plots of the standard deviation for patients (in order top to bottom) of range error with fraction and beam angle, where triangles are 90° and squares are 0°.

To investigate how the random range error changes with both the beam angle and treatment fraction the standard deviation of range errors for each fraction has been plotted for each beam angle. Figure 6 shows the prostate results, where the shape represents a different beam angle, with squares being 0° and triangles being 90°, and each plot is a single patient. Again the *x*-axis is time in terms of treatment fraction, but the *y*-axis is the standard deviation in millimetres for that fraction and beam angle. It can be seen there is a beam angle dependency for most of the patients, with a 0° beam angle resulting in random range errors with a smaller standard deviation.

### 3.1. Population data

All means, Σ’s and *σ*’s for all patients for each beam angle, are shown in tables 2 and 3 and are representative of that treatment site and can used as prior distributions.

**Table 2.** Table of prostate range error in mm for all patients at each beam angle.

Angle	Mean (mm)	Σ (mm)	*σ* (mm)
0	$-0.98$	4.53	5.37
90	2.94	5.94	6.45

All	0.98	5.64	5.91

**Table 3.** Table of head and neck range error in mm for all patients at each beam angle.

Angle	Mean (mm)	Σ (mm)	*σ* (mm)
0	$-1.22$	3.21	4.71
25	$-1.31$	2.97	4.47
70	$-1.91$	3.20	3.93
110	$-2.71$	3.83	4.81
180	$-1.95$	3.48	5.69

All	$-1.82$	3.39	4.72

## 4. Discussion

The data presented has been used to investigate whether this method of WEPL calculation using daily volumetric IGRT could feasibly be used to yield an estimate of residual range error due to inter-fraction motion. Despite limitations in the data set used in this work, this concept can be modified to produce population probability distribution of range changes that occur between fractions. This information would be specific to the anatomical location, immobilisation and method of IGRT. Using this method, probability distributions can be generated and used in either the robust optimisation or at robustness analysis, or even as a method to determine robust beam angles when establishing new planning techniques. By calculating this information on- or off-line, action levels for carrying out dose difference analysis could be established allowing for adaptive planning.

For both treatment sites the mean represents the systematic component of inter-fraction error, whereas the standard deviation represents the random component. For the prostate data the standard deviation of WEPL at each fraction was seen to be larger for 90°. For 90° the standard deviation was greater than that seen for 0° in all except two patients, in one of which the standard deviations of both angles were comparable. The larger range errors seen in the 90° was most likely caused by the fact that the prostate moves independently of bony anatomy and the on set image protocol is to align to a soft tissue match, therefore the density of femoral heads has a large impact on the range error. There will also be an error introduced for the patients that experienced truncation correction. Other groups (Thomas *et al* 2016, Veiga *et al* 2016, Szeto *et al* 2017) have dealt with truncation artefacts through various modality specific methods and each department would need to address their own solutions depending on imaging modality used. Comparing the standard deviations for individual fractions in figure 6 may be misleading. The large standard deviation seen in the prostate population data does not appear to coincide with the individual fraction data. This is due to the large variation in the mean as seen in the heat maps. There have been several papers published indicating that the relative motion of the prostate to the femoral heads is not of dosimetric consequence (Sejpal *et al* 2009, Meyer *et al* 2010, Pugh *et al* 2013). However, these studies have relied upon rigid isocentric shifts and rotations and did not include residual range errors after geometric corrections have been applied. Moteabbed et al(2017) published the first study that investigated the effect of intra-fractional motion for choosing beam angles in prostate proton therapy. In their study, weekly CT scans were deformably registered and dose re-calculated for 10 prostate plans, each planned with both bi-lateral beams and anterior oblique beams. Their results show greater plan robustness for bilateral beams. Though these results appear contradictory to ours, the difference in image guidance protocols used in both studies demonstrate the need to investigate beam angle specific image guidance and registration protocols, in the prostate case for example, matching to bony or soft tissue. Both studies highlight the need to include intra-fractional range changes in robustness analysis due to changes in WEPL to the target from patient motion and residual set-up error.

For the head and neck data the mean range error at each fraction is seen to be closer to zero than that seen in the prostate and there was less variability in the means with fraction. There is a noticeable shift in the mean from zero for several patients with fraction number, which is also seen in the population results. This shows that throughout treatment the WEPL is decreasing. This may be due to slight weight loss and represents a risk of proton overshoot. Our individual fraction results show errors up to, and even exceeding 10 mm may occur, these results are also supported by those published by Liebl *et al* (2014). There is a larger spread in ranges seen for the larger beam angles (70°–180°). This is due to the beam passing through a greater volume of the patient before reaching the target, and travelling through more density heterogeneities. Further investigation using clinical treatment beam angles is required. Due to non- clinical beam angles being used in reality smaller standard deviation for the head and neck population may be expected.

In this work these datasets have been used off-line to quantify inter-fraction range uncertainties. In the case of this study the data sets are used in a way they were not intended for, and it is this reason that gives rise to their limitations for this work. Limited MVCT scanning length of the region of interest has led to missing data, which is seen as gaps in the heat plots. Therefore, a number of fractions could not be analysed, for the prostate patients in particular, it is expected that the values for Σ and *σ* are an overestimate. As well carrying this work out with a larger patient sample, an imaging protocol specific to this goal would be developed to ensure complete data sets. Further work would also be to provide suitable correction strategies for determining the systematic error would be to identify those errors unlikely to have occurred by chance, and apply a correction as suggested by Greener (2003). This work has not attempted to investigate the systematic range error due to HU calibration.

Ultimately, it has been shown how the use of daily volumetric IGRT can be used to quantify residual random range error with inter-fraction motion for individual patient cases, and to collect population data, as a proof of principle. Our results highlight that residual range errors after daily IGRT need greater investigation through producing site specific models. Future work will be to use Cone Beam CT, deformable image registration, clinical treatment plans and larger data sets.

## 5. Conclusion

There is a need in proton therapy planning to be able to quantify the random range uncertainty to ensure plan robustness. A method of using daily volumetric imaging from patients previously treated with conventional radiotherapy to quantify range uncertainty from inter-fraction motion is described. A greater understanding in range uncertainty better informs the planner on how best to balance the trade-off between plan conformality and robustness in proton therapy.

This work demonstrates how the use of daily imaging data sets from previously treated patients can be used to quantify the random range uncertainty in the WEPL within the CTV caused by inter-fraction motion. This method has potential for use in investigating inter-fraction range uncertainty in proton therapy. The method has been used for two treatment sites and several beam angles. This concept can be extended to investigate the change in range between distal edge of CTV and proximal edge or OARs in the beam direction as a measure of robustness.
